# Cross-trait analyses identify shared genetics between migraine, headache, and glycemic traits, and a causal relationship with fasting proinsulin

**DOI:** 10.1007/s00439-023-02532-6

**Published:** 2023-02-20

**Authors:** Md Rafiqul Islam, The International Headache Genetics Consortium (IHGC), Dale R. Nyholt

**Affiliations:** Statistical and Genomic Epidemiology Laboratory, School of Biomedical Sciences, Faculty of Health and Centre for Genomics and Personalised Health, Queensland University of Technology, Brisbane, QLD Australia

## Abstract

The co-occurrence of migraine and glycemic traits has long been reported in observational epidemiological studies, but it has remained unknown how they are linked genetically. We used large-scale GWAS summary statistics on migraine, headache, and nine glycemic traits in European populations to perform cross-trait analyses to estimate genetic correlation, identify shared genomic regions, loci, genes, and pathways, and test for causal relationships. Out of the nine glycemic traits, significant genetic correlation was observed for fasting insulin (FI) and glycated haemoglobin (HbA1c) with both migraine and headache, while 2-h glucose was genetically correlated only with migraine. Among 1703 linkage disequilibrium (LD) independent regions of the genome, we found pleiotropic regions between migraine and FI, fasting glucose (FG), and HbA1c, and pleiotropic regions between headache and glucose, FI, HbA1c, and fasting proinsulin. Cross-trait GWAS meta-analysis with glycemic traits, identified six novel genome-wide significant lead SNPs with migraine, and six novel lead SNPs with headache (*P*_meta_ < 5.0 × 10^–8^ and *P*_single-trait_ < 1 × 10^–4^), all of which were LD-independent. Genes with a nominal gene-based association (*P*_gene_ ≤ 0.05) were significantly enriched (overlapping) across the migraine, headache, and glycemic traits. Mendelian randomisation analyses produced intriguing, but inconsistent, evidence for a causal relationship between migraine and headache with multiple glycemic traits; and consistent evidence suggesting increased fasting proinsulin levels may causally decrease the risk of headache. Our findings indicate that migraine, headache, and glycemic traits share a common genetic etiology and provide genetic insights into the molecular mechanisms contributing to their comorbid relationship.

**Supplementary Information:**

The online version contains supplementary material available at 10.1007/s00439-023-02532-6.

## Introduction

Migraine is a debilitating neurological disorder that affects about 15% of the global population and was first described as a ‘hypoglycemic headache’ in 1935 (Gray and Burtness 1935). A high prevalence of glycemic traits such as insulin resistance (IR), hyperinsulinemia, hypoglycaemia, hyperglycaemia, and type 2 diabetes (T2D) is associated with migraine and headache (Cavestro et al. 2007; Fagherazzi et al. 2019; Gross et al. 2019; Rainero et al. 2005; Sacco et al. 2014; Zhang et al. 2020), which, along with the increased risk of cardiovascular diseases (Daghals et al. 2022; Guo et al. 2020; Malik et al. 2015), contributes significantly to shorter life expectancy. Previous epidemiologic studies investigating the relationship between fasting glucose (FG), fasting insulin (FI), and glucose tolerance with migraine risk have produced conflicting findings (Islam and Nyholt 2022; Shaw et al. 1977; Siva et al. 2018). In addition, several studies have investigated the increased comorbidity of migraine and glycemic traits, focusing on the dysregulation of glucose (Hufnagl and Peroutka 2002; Zhang et al. 2020) or dysglycaemia in migraine patients as possible outcome of disturbed metabolism mainly due to impaired glucose-insulin metabolism (Gross et al. 2019).

Dysglycaemia is a well-established migraine risk factor that may have a role in the etiology of migraine and headache disorders, given the significance of glycemic regulation in the brain and the fact that it has well-known systemic effects (Mergenthaler et al. 2013); nevertheless, the role of dysglycaemia in the brain is sometimes overlooked. Additionally, insulin signalling alterations may be related to migraine disorders since insulin plays a vital role in synaptic plasticity, neurotransmission, and neuroinflammation in the central nervous system (Del Moro et al. 2022; Duarte et al. 2012). These findings imply that metabolic and glycemic dysregulations may be integral to the pathophysiology of migraine. The phenotypic and pathophysiological similarities observed between migraine and glycemic traits have led us to hypothesise that a shared genetic basis may contribute to both conditions (Islam and Nyholt 2022). In addition, we recently reported that shared genetic factors between migraine and headache with T2D strongly reflect their epidemiological observational relationships (Md Rafiqul Islam et al. 2022). Although lower FG, increased FI, decreased glucose tolerance, and increased IR have all been associated with migraine in observational studies (Shaw et al. 1977; Siva et al. 2018; Zhang et al. 2020), it is still unknown whether these associations are genetically correlated.

An observational relation between two traits may denote hereditary or environmental influences or a combination of the two. There may be an overlap in the causative genes and pathways underlying the multiple risk factors shared by two complex traits. Hence, clustering genetic variants and genes shared by multiple traits may shed light on the underlying biology of their comorbidity. Such common genetic elements can be examined using a cross-trait genome-wide analysis approach. Based on these observations, an in-depth study of shared genetics by exploring the relationship between migraine and glycemic traits is warranted. Therefore, in the present study, we conducted genetic analyses leveraging GWAS summary statistics to investigate the shared genetic contributions between migraine and headache with glycemic traits.

## Materials and methods

### Study design

**Fig. 1:**
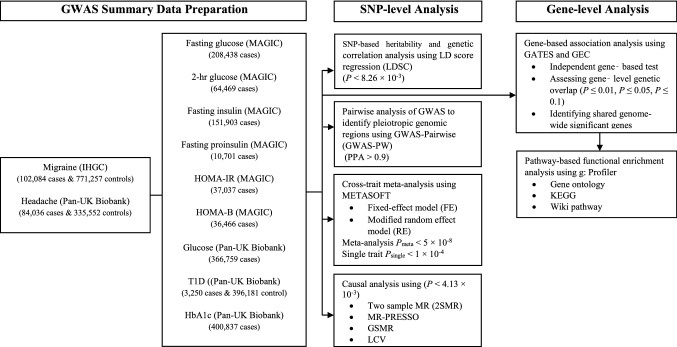
Overall research plan and brief overview of the analysis. [*IHGC* International headache genetic consortium, *MAGIC* Meta-Analyses of Glucose and Insulin-related traits Consortium, *Pan-UK Biobank* Pan-ancestry genetic analysis of the UK Biobank, *PPA* Posterior probabilities of association, *LD* Linkage disequilibrium, *GATES* Gene-Based Association Test Using Extended Simes Procedure, *GEC* Genetic type 1 error calculator, *GWAS* Genome-wide association studies, *KEGG* Kyoto Encyclopedia of Genes and Genomes, *LCV* Latent causal variable, *MR* Mendelian randomization, *MR-PRESSO* Mendelian randomisation pleiotropy residual sum and outlier, *GSMR* Generalised summary data-based Mendelian Randomisation, *SNP* Single-nucleotide polymorphism, *T1D* Type 1 diabetes, *HbA1c* Glycated haemoglobin, *HOMA-B* homeostatic model assessment of β-cell function, *HOMA-IR* homeostatic model assessment of insulin resistance]

Figure 1 summarises the overall study design and workflow. This study has three analytical stages. The first stage is collecting and preparing GWAS summary statistics of migraine, headache, and glycemic traits of European descent to perform different analytical methods. The second stage is SNP-level analyses to identify genetic overlap, novel loci, and causal associations between migraine and headache with glycemic traits. The third stage is gene-level analyses to investigate the gene-based association and shared genes, and finally, pathway analyses using shared genes between migraine and headache with glycemic traits.

### GWAS summary statistics for migraine

We obtained GWAS summary statistics for migraine, comprising 102,084 migraine patients and 771,257 controls of European ancestry (Hautakangas et al. 2022). Full genotyping and phenotyping procedures are detailed in the original publication (Hautakangas et al. 2022).

### GWAS summary statistics for headache

In our analysis, we obtained the publicly available Pan-UKBB (Pan-ancestry genetic analysis of the UK Biobank) (Pan-UKB team 2020) GWAS summary statistics of European ancestry for headache experienced last month. In our research, headache patients were those who had a headache within the last month that interfered with their usual activities (Pan-UKB team 2020). We used phenocode 6159 for “headache” (a total of 400,837 individuals, comprising 84,036 headache cases and 335,552 non-headache controls). Briefly, between 2005 and 2010, the UK Biobank prospectively recruited 500,000 individuals of various ancestries living in the United Kingdom between the ages of 40 and 69 and conducted a genotypic and phenotypic evaluation (Bycroft et al. 2018). Genotyping, quality control, and access to GWAS summary statistics are available at https://pan.ukbb.broadinstitute.org.

### GWAS summary statistics for glycemic traits

Publicly available GWAS summary statistics for glucose, glycated haemoglobin (HbA1c), and Type 1 diabetes (T1D) were obtained from the Pan-UKBB. We used phenocode 250.1 for “T1D” (total 399,431 individuals, comprising 3,250 cases and 396,431 controls), biomarker 30,750 for “HbA1c” (total 400,837 individuals), and biomarker 30,740 for “glucose” (total 366,759 individuals) to identify and access the GWAS summary statistics from the Pan-UKBB.

We collected publicly available GWAS summary statistics for fasting glucose (FG), fasting insulin (FI), and 2-h glucose after an oral glucose challenge (2-h glucose) from the meta-analyses of glucose and insulin-related traits consortium (MAGIC) (https://magicinvestigators.org/), comprising a total of 281,416 individuals with more than 70% European descent (Chen et al. 2021). The FG, FI, and 2-h glucose GWAS comprised 208,438, 151,903, and 64,469 participants, respectively. We also obtained MAGIC GWAS summary statistics for homeostatic model assessment of insulin resistance (HOMA-IR), HOMA of pancreatic beta-cell function (HOMA-B), and fasting proinsulin (Dupuis et al. 2010; Strawbridge et al. 2011). The HOMA-B and HOMA-IR GWAS comprised 46,186 individuals without diabetes, and the fasting proinsulin GWAS comprised 10,701 non-diabetic European individuals.

### Imputation of GWAS summary statistics

To improve SNP overlap across some of the datasets, we used the recently published RAISS approach (Julienne et al. 2019) to impute missing Z-scores for the GWAS summary statistics imputed on the HapMap 2 reference panel. First, using PLINK 1.9 (Chang et al. 2015), an LD-correlation matrix was prepared for 1,703 predefined LD-independent regions (Berisa & Pickrell 2016) from the 1000 Genomes Project European reference panel. Next, Z-scores were derived using summary statistics from HOMA-B, HOMA-IR, and fasting proinsulin GWAS. After that, RAISS was run using the default settings. Then, for imputed SNPs, effect sizes (beta) and standard errors of the effect estimate (SE) were calculated using the equation reported in the previously published study (Zhu et al. 2016). Finally, we filtered imputed SNPs with *R*^2^ < 0.6 to improve the quality of the results.

### Genetic correlation analysis

SNP-based bivariate genetic correlations (*r*_g_) were calculated using the LD score regression (LDSC) software (Bulik-Sullivan et al. 2015; Bulik-Sullivan et al. 2015) to investigate the genetic overlap of migraine with glycemic traits and headache with glycemic traits. The estimated range of the LDSC *r*_g_ is from − 1 to 1, where − 1 indicates an absolute negative genetic correlation and 1 indicates an absolute positive genetic correlation. We utilised pre-computed estimates of LD scores from the HapMap3 European reference panel of ~ 1.2 million common SNPs. One of the main strengths of LDSC is that it can estimate the genetic correlation when there is a sample overlap since sample overlap only affects the LDSC intercept and not the slope from which the *r*_g_ is estimated. In addition, cross-trait genetic covariance intercepts (indicating sample overlap) were constrained to zero if they were not significantly different from zero. We examined nine glycemic traits in this study; however, these are correlated to a certain degree. Hence, we estimated the effective number of independent traits using matrix spectral decomposition (matSpD) (Nyholt 2004) of the pairwise *r*_g_ matrix (Supplementary Table S4). MatSpD estimated that the nine glycemic traits were equivalent to 6.1849 independent traits. Therefore, to adjust for multiple testing, we considered a genetic correlation finding study-wide significant in the LDSC analysis at *P* < 8.26 × 10^–3^ (0.05/6.1849) and nominally significant when *P* < 0.05.

### Pairwise analysis of GWAS

We used pairwise GWAS analysis (GWAS-PW) (Pickrell et al. 2016) to identify genomic regions shared by migraine and glycemic traits, and by headache and glycemic traits. This technique divides the entire genome into 1703 LD-independent regions (average size of 1.5 Mb) (Berisa and Pickrell 2016) and estimates the posterior probability association (PPA) for four models using a Bayesian statistical model. In models 1 and 2, the region is assumed to harbour a genetic variant associated with only trait 1 or trait 2, respectively. Model 3 implies that the region contains a genetic variant associated with both traits (PPA3), while model 4 assumes that the region harbours two independent variants distinctly associated with each trait (Pickrell et al. 2016). Genomic regions with a model 3 PPA3 > 0.9 were considered to harbour a significant pleiotropic effect, while regions with a PPA3 > 0.5 harbour a suggestive pleiotropic effect.

### Cross-trait meta-analysis between migraine and headache with glycemic traits

To detect shared SNPs and loci, we conducted a cross-trait meta-analysis between migraine and glycemic traits, and between headache and glycemic traits. We used the fixed effect (FE) and the modified random effects (RE2) models within the METASOFT software (Han and Eskin 2011). The FE model is based on the fixed-effect meta-analysis method and is most effective when genetic effect sizes are homogeneous. However, genetic effect sizes are unlikely to be homogenous when analysing multiple traits. Thus, the RE2 model (Han and Eskin 2011) was used as an extension of the FE method, which is more robust and can accommodate heterogeneous effects for different traits. SNPs and loci that became genome-wide significant after meta-analysis (*P*_meta_ < 5 × 10^–8^) but were not genome-wide significant in the individual trait GWAS before meta-analysis (5 × 10^–8^ < *P*_single-trait_ < 0.05) were considered potentially novel.

Additionally, we used the METASOFT m-value method to assess the likelihood of novel SNP effects existing in the individual traits (Han and Eskin 2012). The m-value reflects the posterior probability that the effect is present in each trait of the cross-trait meta-analysis. M-values larger than 0.9 denote the presence of the effect, m-values less than 0.1 denote the absence of the effect, and m-values between 0.1 and 0.9 denote ambiguity regarding the presence of the effect in each trait GWAS (Han and Eskin 2012).

### Characterisation of independent novel lead SNPs

We used the FUMA web tool (Watanabe et al. 2017) to clump the association results from our cross-trait meta-analysis of migraine and glycemic traits, and headache and glycemic traits to identify independent lead SNPs (*r*^2^ < 0.1). We began by identifying significant independent SNPs based on their cross-trait meta-analysis *P-*value (*P* < 5 × 10^–8^) and independence from one another (*r*^2^ < 0.6) within a 1 Mb frame (Md Rafiqul Islam et al. 2022). Next, lead SNPs were identified using a subset of the significant independent SNPs. Using the same 1 Mb frame, significant independent SNPs in LD with one another at (*r*^2^ < 0.1) were classified as the lead SNP. The European ancestry 1000 Genomes Project reference panel within FUMA was used to calculate all LD information (Auton et al. 2015). More information on the LD clump approach can be found on the FUMA website (http://fuma.ctglab.nl/) (Watanabe et al. 2017). Each trait’s genome-wide significant SNPs from the original GWAS were classified as known lead variants. Lead SNPs in our cross-trait meta-analyses that were in LD (*r*^2^ > 0.1) with an original GWAS trait SNP were considered to be within a known locus and thus excluded (Md Rafiqul Islam et al. 2022). The remaining significant lead SNPs from our cross-trait meta-analyses were identified as putative novel lead SNPs. If putative novel lead SNPs or SNPs in LD (*r*^2^ > 0.1) mapping to the same gene(s) did not overlap with any previously reported loci in the GWAS catalog (https://www.ebi.ac.uk/gwas/; search date: 8 January 2023) for the same traits used in the meta-analysis, we identified it as a novel locus. To identify novel SNPs with strong evidence for association to both migraine and a glycemic trait, we highlight the novel lead SNPs with a genome-wide significant association in the cross-trait meta-analysis (*P*_meta_ < 5 × 10^–8^) and suggestive association in the individual migraine and glycemic trait GWAS (*P*_single trait_ < 1 × 10^–4^).

### Examining causal relationships between migraine and headache with glycemic traits

#### Mendelian randomisation

We used two-sample Mendelian randomisation (2SMR) approaches to test for a causal relationship (vertical pleiotropy) between the genetic liability to glycemic traits on migraine and headache, as well as the reverse of causal effects of genetic liability to migraine and headache on glycemic traits. Inverse-variance weighted (IVW) model was used as the primary method (Burgess et al. 2013). In the exposure GWAS, SNPs with GWAS *P* < 5 × 10^–8^ that are LD-independent were employed as instrumental variables (IVs). The IVW model has the drawback that even a single invalid IV might cause the overall estimate to be biased (Burgess et al. 2013). Therefore, we used four sensitivity methods to test its validity. First, we applied the weighted median model that can provide a reliable causal estimate even when less than half of the weight of the IVs does not meet the MR assumptions (Bowden et al. 2016). The second method used the MR-Egger model, which tests explicitly for horizontal pleiotropy (Bowden et al. 2015), although the MR-Egger model has lower statistical power compared to the IVW model. Third, the MR pleiotropy residual sum and outlier (MR-PRESSO) (Verbanck et al. 2018) method was used, which comprised of three tests: (i) global test to assess whether horizontal pleiotropy exists or not; (ii) outlier test to remove potential outliers to control horizontal pleiotropy, and (iii) distortion test to examine whether the causal estimate is significantly different from before and after the removal of an outlier. Finally, we used generalised summary data-based Mendelian randomisation (GSMR) (Zhu et al. 2018) to test for a causal relationship between migraine and headache with glycemic traits. The default *P* ≤ 5 × 10^–8^ and *r*^2^ < 0.05 thresholds were used to choose LD-independent IVs, although because the GSMR guidelines advise utilising at least ten LD-independent IVs to produce reliable results, a *P* ≤ 1 × 10^–5^ threshold was applied when fewer than 10 SNPs met the default level. The HEIDI (heterogeneity in the dependent instrument) outlier procedure is used in the GSMR analysis to remove SNPs that have a pleiotropic effect (Zhu et al. 2018). Therefore, we set the HEIDI outlier detection analysis’s *P*-value threshold to 0.01, eliminating 1% of SNPs by chance even when there are no pleiotropic effects. The MR analyses in this study were performed using the ‘TwoSampleMR’ v0.5.6 (Hemani et al. 2018), ‘MR-PRESSO’ v1.0 packages (Verbanck et al. 2018), and the GSMR analysis built into the GCTA v1.93.2 software (Yang et al. 2011). Multiple testing correction was applied to all MR tests, and a *P* value threshold of *P* < 4.13 × 10^–3^ (0.05/6.1849/2, where 6.1849 indicates the total number of independent glycemic traits and 2 represents the inclusion of both forward and reverse experiments in the MR analysis) was deemed study-wide significant, and *P* < 0.05 was considered nominally significant.

#### Latent causal variable model

We utilised a latent causal variable (LCV) model (O’Connor and Price 2018) to examine whether a genetic correlation demonstrates a causal relationship. The LCV model calculates a genetic causality proportion (GCP) between migraine and headache with glycemic traits. LCV considers that a latent variable mediates the genetic correlation between the traits and assesses the strength of each trait’s correlation with this latent variable (O’Connor and Price 2018). Weak GCP values near zero for genetically correlated traits suggest no genetic causality, and their association is likely influenced by horizontal pleiotropy. In contrast, a GCP of one indicates complete genetic causality. We used the suggested threshold (GCP > 0.6) for partial genetic causality because it has been shown in simulations to guard against false positives adequately. As recommended by the LCV developers, the major histocompatibility complex (MHC) region was excluded due to its complicated LD structure, and only SNPs with a minor allele frequency (MAF) > 0.05 were kept in the GWAS summary statistics (O’Connor and Price 2018). Analogous to the LDSC analyses (Bulik-Sullivan et al. 2015), HapMap3 SNPs outside the MHC region (MAF > 0.05) were used to harmonise (‘munge’) all trait GWAS summary statistics prior to the LCV analysis, and we used the LD scores for HapMap3 SNPs (MHC region omitted) from the 1000 Genomes Project European reference panel (Abecasis et al. 2012; Consortium 2010).

### Gene-based association analysis

#### Genes-based test

Gene-level association analysis has an advantage over individual SNP-based studies in that it can integrate the effects of multiple SNPs and may provide greater power for identifying genetic risk variants for complex traits (Zhao et al. 2016). We used the Gene-Based Association Test Using Extended Simes Procedure (GATES) test (Li et al. 2011), integrated into the Fast ASsociation Tests (FAST) package (Chanda et al. 2013), to perform a gene-based association test using SNPs overlapping migraine, headache, and glycemic trait GWAS summary statistics. *P*-values were calculated using SNPs annotated to protein-coding genes to determine the association with migraine, headache, and glycemic traits. NCBI build 37 (The human genome version 19) was used to determine the locations and boundaries of the genes, and the 1000 Genomes Project European reference panel was utilised to estimate LD (Md Rafiqul Islam et al. 2022). Using NCBI 37 gene coordinate information, SNPs were mapped to 19,418 protein-coding genes, and SNPs found within 10 kb of each gene were assigned to that gene (Md Rafiqul Islam et al. 2022). GATES computes a gene-based *P*-value from the *P*-values of SNPs assigned to the same gene. The GATES test has the advantage of requiring only GWAS summary statistics and a suitable LD reference. In addition, GATES estimates empirical significance without the need for permutation or simulation and can effectively control the type 1 error rate (due to testing multiple SNPs in a gene) regardless of gene size and LD pattern among SNPs (Li et al. 2011). Finally, the minSNP gene-based test was applied, which adjusts the minimal *P*-value assigned to the gene by the effective number of independent SNPs associated with the gene (Chanda et al. 2013; Md Rafiqul Islam et al. 2022).

#### Independent gene-based test

Gene-based association results may be correlated across neighbouring genes due to LD between the most significant SNP (‘best SNP’) assigned to each gene. Thus, the genetic type I error calculator (GEC) (Li et al. 2012) was employed to estimate the effective number of independent genes (i.e., the number of independent gene-based tests) by examining the LD between the most significant SNP assigned to each gene and providing the correct type 1 error rate. GEC accounts for multiple testing and appropriately controls the type 1 error rate (Li et al. 2012). This analysis was carried out using the GEC software, which has previously been used in other investigations (Adewuyi et al. 2021; Zhao and Nyholt 2017). The GEC method first partitioned the input SNPs into independent LD blocks (*r*^2^ < 0.1), and then for each LD block, it conducted an eigenvalue analysis of the correlation matrix to calculate the effective number of independent SNPs (Li et al. 2012). As GEC input, we used the ‘best-SNPs’ (providing the minimal *P*-value for each gene) found in our gene-based analysis. We estimated the effective number of independent genes for each GWAS trait individually.

#### Gene-based genetic overlap test

We tested whether the proportion of associated genes overlapping migraine and glycemic traits and headache and migraine was more than expected by chance at three different nominal *P*-values thresholds (gene with *P*_gene_ ≤ 0.01, *P*_gene_ ≤ 0.05, and *P*_gene_ ≤ 0.1). The number of genes overlapping both traits at each of the three *P*-value levels was first referred to as the raw number of overlapping genes. Then, we calculated the effective number of independent overlapping genes using independent gene-based analysis to determine whether the proportions of overlapping genes were more than expected by chance (Adewuyi et al. 2020; Zhao et al. 2016). The migraine or headache GWAS was assigned as the ‘discovery’ dataset, and the glycemic traits GWAS was assigned as the ‘target’ dataset. The effective number of genes with *P*-values less than the threshold in the discovery and target datasets was used to define this study’s observed number of overlapping genes (Zhao and Nyholt 2017). The observed proportion of overlapping genes was calculated by dividing the observed effective number of overlapping genes by the effective number of genes in the discovery dataset with a *P*-value less than the threshold (Zhao et al. 2016). The effective number of genes in the target dataset with a *P*-value less than the threshold divided by the total effective number of genes in the target dataset represented the expected proportion of overlapping genes (Zhao et al. 2016). To assess the statistical significance at the three *P-*value thresholds, we performed an exact binomial test to compare the proportion of observed and expected overlapping independent genes. We examined whether the proportion of overlapping genes was higher than expected by chance. Finally, we conducted a cross-trait gene-based association meta-analysis to find significant shared genes associated with migraine and glycemic traits, and headache and glycemic traits. We employed Fisher’s combined *P*-value (FCP) method to combine gene-based association *P*-values across two traits. Recent research (Adewuyi et al. 2020, 2021; Zhao et al. 2016) employed this gene-based strategy to demonstrate gene-based pleiotropy across multiple traits.

### Pathway-based functional enrichment analysis of overlapping genes

We used the g:GOst webtool (Raudvere et al. 2019; Reimand et al. 2016) implemented in the g-profiler software to investigate the enrichment of shared genes in the Gene Ontology (GO) biological process, Kyoto Encyclopedia of Genes and Genomes (KEGG), Reactome, and Wiki Pathways. The genes overlapping migraine and glycemic traits, and headache and glycemic traits at *P*_gene_ < 0.05 were utilised as input to identify pathways. However, we used genes overlapping at *P*_gene_ < 0.01 between migraine with glucose and HbA1c, and between headache and HbA1c to keep the number of genes less than the recommended maximum of 1000. Using the default and suggested ‘g:SCS algorithm’, an adjusted *P*-value (*P*_adj_ < 0.05) was calculated that accounts for multiple testing (Raudvere et al. 2019). Furthermore, the functional category’s term sizes were lowered to values between 5 and 350. We kept all advanced settings at their default values in our analysis.

## Results

### Genetic correlations between migraine and headache with glycemic traits

Our genetic correlation analyses reflect the relationships between migraine and headache with glycemic traits. We found migraine (*r*_g_ = 0.08, *P* = 4.07 × 10^–5^) and headache (*r*_g_ = 0.09, *P* = 5.0 × 10^–4^) genetically correlated with FI. Likewise, there were significant genetic correlations between migraine (*r*_g_ = 0.05, *P* = 5.0 × 10^–4^) and headache (*r*_g_ = 0.08, *P* = 2.58 × 10^–5^) with HbA1c. Additionally, migraine (*r*_g_ = − 0.13, *P* = 4.59 × 10^–2^) and headache (*r*_g_ = − 0.20, *P* = 1.25 × 10^–2^) demonstrated a significant genetic correlation with fasting proinsulin, although this relationship was not significant after adjusting for multiple testing. Furthermore, 2-h glucose (*r*_g_ = 0.07, *P* = 6.63 × 10^–3^) and T1D (*r*_g_ = 0.12, *P* = 3.16 × 10^–2^, not significant after adjusting for multiple testing) both produced evidence for genetic correlation with migraine, but not with a headache. There was no evidence of a genetic correlation between migraine and headache with glucose, FG, HOMA-B, and HOMA-IR (all *P* > 0.05) (Table 1). Supplementary Table S2 displays the SNP-based heritability of migraine, headache, and glycemic traits on the observed scale using the described GWAS summary statistics. Although the LDSC heritability (*h*^2^) and *r*_g_ analyses were robust to potential sample overlap, to aid in the interpretation of downstream analyses, we examined the LDSC genetic covariance intercept (gcov_int). For migraine, the gcov_int values were all relatively small, with only 2-h glucose (*P* = 0.016) and HOMA-IR (*P* = 0.044) significantly different from zero, and these were not significant after adjusting for multiple testing. Similarly, the gcov_int values for headache were small, with only glucose (*P* = 0.005), proinsulin (*P* = 0.021), and T1D (*P* = 0.003) being significantly different from zero, and only glucose and T1D significant after adjusting for multiple testing. The gcov_int results (Supplementary Table S3) suggest no substantial sample overlap between migraine and headache with each of the glycemic traits, and the cross-trait SNP meta-analyses were unlikely to be confounded by sample overlap.

**Table 1:** SNP-based genetic correlation results for migraine and headache with glycemic traits based on LD score regression analysis

Trait 1	Trait 2	Rg	SE	*P*
Migraine	FG	0.0005	0.016	9.75 × 10^–1^
	2-h glucose	0.07	0.026	6.60 × 10^–3^
	Glucose	0.02	0.015	2.43 × 10^–1^
	FI	0.08	0.019	4.07 × 10^–5^
	Proinsulin	− 0.13	0.067	4.95 × 10^–2^
	HOMA-IR	− 0.07	0.054	1.72 × 10^–1^
	HOMA-B	0.02	0.031	5.63 × 10^–1^
	T1D	0.12	0.054	3.16 × 10^–2^
	HbA1c	0.05	0.015	5.00 × 10^–4^
Headache	FG	0.003	0.019	8.79 × 10^–1^
	2-h glucose	0.02	0.031	4.96 × 10^–1^
	Glucose	0.03	0.030	3.86 × 10^–1^
	FI	0.09	0.026	5.00 × 10^–4^
	Proinsulin	− 0.20	0.080	1.25 × 10^–2^
	HOMA-IR	0.05	0.050	3.20 × 10^–1^
	HOMA-B	0.06	0.044	1.44 × 10^–1^
	T1D	0.07	0.085	4.17 × 10^–1^
	HbA1c	0.08	0.019	2.58 × 10^–5^

*Rg* genetic correlation estimate, *SE* standard error of genetic correlation estimate, *SNP* single-nucleotide polymorphism, *LD* linkage disequilibrium, *T1D* Type 1 diabetes, *FG* Fasting glucose, *FI* Fasting insulin, *Proinsulin* Fasting proinsulin, *HOMA-B* homeostatic model assessment of β-cell function, *HOMA-IR* homeostatic model assessment of insulin resistance, *2-h glucose* 2-h glucose after an oral glucose challenge, *HbA1c* glycated haemoglobin

### Pleiotropic genomic regions influencing migraine, headache, and glycemic traits

In our GWAS-PW analysis, we identified eight (PPA3 > 0.9) and 17 (PPA3 > 0.5) genomic regions with a shared association between migraine and any of the four glycemic traits (FG, FI, HbA1c, and glucose) (Supplementary Table S5). The findings from the GWAS-PW study of migraine and glycemic traits, and headache and glycemic traits are described in Table 2. A highly pleiotropic locus at chromosome 14q32.12-q32.13 was found to drive the associations between migraine and FI and between migraine and HbA1c. Five loci (1q32-q23.1, 2q34, 6q25.3-q26, 14q32.12-q32.13, and 17p13.3-13.2) were associated significantly with HbA1c and migraine, and three of these loci were common with those identified between headache and HbA1c (Table 2). GWAS-PW analyses of migraine and FG identified significant pleiotropic loci at chromosomes 9q34.13-q34.20 and 19q13.32. No significant (PPA3 > 0.9) pleiotropic loci were identified between migraine with other glycemic traits (glucose, 2-h glucose, fasting proinsulin, HOMA-B, HOMA-IR, and T1D). GWAS-PW analyses with headache found 12 (PPA3 > 0.9) and 26 (PPA3 > 0.5) genomic regions to be significantly shared across six glycemic traits (glucose, FG, FI, fasting proinsulin, HbA1c, and T1D) (Supplementary Table S5). Among the 12 pleiotropic loci with PPA3 > 0.9 between headache and glucose, FI, fasting proinsulin, and HbA1c, three (1q32-q23.1, 2q34, and 14q32.12-q32.13) overlapped with loci identified in our GWAS-PW analysis of migraine and glycemic traits (Table 2). We found one pleiotropic locus between headache and glucose (12p13.32) and fasting proinsulin (11q13.4), and several pleiotropic loci between headache and FI (8p23.1, 9q33.1, and 14q32.12-q32.13) and HbA1c (1q32-q23.1, 2q34, 5q31.1, 7p22.3, 8p23.1-p22, 12p13.32, and 14q32.12-q32.13).

**Table 2:** Genomic regions associated with migraine and glycemic traits and with headache and glycemic traits (posterior probability of association [PPA3] > 0.9) using GWAS-PW analysis

Trait 1	Trait 2	Region	Locus	Chr	Start bp	End bp	PPA1	PPA2	PPA3	PPA4	Overlapping genome-wide significant genes in the indicated region for both traits (*P*_single trait_ < 0.05 and *P*_meta_ FCP < 3.65 × 10^–06^)
Migraine	FG	1005	9q34.13-q34.20	chr9	135298917	137040737	0.00	0.00	0.92	0.08	*ABO, MED22, RPL7A, SURF1, SURF2, BRD3
		1609	19q13.32	chr19	46102697	47148853	0.00	0.00	0.95	0.05	GIPR, SNRPD2, *QPCTL, *FBXO46, *BHMG1, *SIX5, *DMPK, *DMWD, *RSPH6A, *SYMPK, *FOXA3
Migraine	FI	1370	14q32.12-q32.13	chr14	94325812	95750857	0.02	0.00	0.98	0.00	*SERPINA2, SERPINA1*
Migraine	HbA1c	78	1q32-q23.1	chr1	156336133	158027330	0.00	0.00	0.98	0.02	RHBG, *C1orf61, *MEF2D, IQGAP3, *NTRK1
		256	2q34	chr2	209941529	212379238	0.01	0.00	0.98	0.00	*CPS1
		734	6q25.3-q26	chr6	160581374	162169452	0.00	0.01	0.90	0.09	*SLC22A3, LPA
		1370	14q32.12-q32.13	chr14	94325812	95750857	0.00	0.00	1.00	0.00	*SERPINA2, *SERPINA1
		1485	17p13.3-13.2	chr17	1929074	3701935	0.00	0.00	0.91	0.09	*DPH1, *OVCA2, *HIC1, *SMG6, *SRR, TSR1, SGSM2
Headache	Glucose	1182	12p13.32	chr12	3677111	4417127	0.00	0.00	1.00	0.00	*CCND2
Headache	FI	852	8p23.1	chr8	7154036	9154609	0.00	0.00	1.00	0.00	*CLDN23, *MFHAS1, ERI1, *PPP1R3B
		995	9q33.1	chr9	117922331	121321501	0.06	0.00	0.92	0.02	*ASTN2
		^a^1370	14q32.12-q32.13	chr14	94325812	95750857	0.00	0.00	1.00	0.00	*SERPINA2, SERPINA1*
Headache	Proinsulin	1134	11q13.4	chr11	70926665	72284569	0.00	0.00	0.99	0.01	*–*
Headache	HbA1c	^a^78	1q32-q23.1	chr1	156336133	158027331	0.00	0.00	0.99	0.01	*C1orf61, *MEF2D, NTRK1
		^a^256	2q34	chr2	209941529	212379410	0.00	0.01	0.92	0.04	*CPS1
		600	5q31.1	chr5	132139649	134777267	0.00	0.00	0.97	0.03	*HSPA4
		745	7p22.3	chr7	1353067	2062096	0.00	0.00	0.95	0.05	*MAD1L1
		856	8p23.1-p22	chr8	11279164	13491594	0.00	0.00	0.99	0.00	*BLK
		1182	12p13.32	chr12	3677111	4417127	0.00	0.00	0.99	0.00	*CCND2
		^a^ 1370	14q32.12-q32.13	chr14	94325360	95750857	0.00	0.00	1.00	0.00	*ITPK1, *SERPINA2, *SERPINA1

*T1D* Type 1 diabetes; FG: Fasting glucose; FI: Fasting insulin; HbA1c: Glycated haemoglobin; Chr: chromosome; Start bp: Start base pair; End bp: End base pair; PPA1: posterior probability for model 1 (association only to trait 1); PPA2: posterior probability for model 2 (association only to trait 2); PPA3: posterior probability for model 3 (shared association to both traits); PPA4: posterior probability for model 4 (two distinct associations of both trait). ^a^Genomic regions are common in both migraine and headache with glycemic traits. *Genes have top SNPs with single-trait *P* < 1 × 10^–5^ for migraine, headache, and respective glycemic trait

### Cross-trait meta-analysis between migraine and headache with glycemic traits

The cross-trait meta-analysis combining migraine and glycemic traits and headache and glycemic traits found 44 (42 are novel for both traits) and 40 (33 are novel for both traits) potential novel independent lead SNPs (*P*_meta_ < 5 × 10^–8^ and *P*_single-trait_ < 0.05), respectively (Supplementary Tables S6 and S7). We highlight the lead SNPs having genome-wide significance in the cross-trait meta-analysis (*P*_meta_ < 5 × 10^–8^) and suggestive association in the individual trait GWAS (*P*_single-trait_ < 1 × 10^–4^). Seven of the 44 putative novel lead SNPs between migraine and glycemic traits met this criterion (Table 3). According to the results of posterior probability (m-value) analysis, all the reported lead SNPs discovered appeared to be associated with both migraine and the respective glycemic trait (i.e., the m-value for both headache and glycemic traits was equal to one). These seven putative novel lead SNPs were mapped to the nearest protein-coding genes of *MANBA*, *RCCD1*, *ROBO1*, *KDM4A*, *ADAMTS9*, *KCNK16*, and *MYBPC3*. Six of these lead SNPs or loci are novel for both migraine and glycemic traits as they were not previously reported to be associated with these traits, with one locus (*MANBA*) being novel for migraine but not FG. The strongest signal was found on chromosome 4 in the *MANBA* region (lead SNP rs223482, *P*_meta_ = 5.85 × 10^–11^). The second strongest signal was found on chromosome 3, closest to the *ADAMTS9* gene (lead SNP rs4611812, *P*_meta_ = 1.54 × 10^–10^).

**Table 3:** Cross-trait meta-analysis results for migraine and glycemic traits GWAS (*P*_meta_ < 5 × 10^–8^ and single trait *P* < 10^–4^)

Lead SNP	CHR	BP	EA	NEA	RE2 Meta-analysis			Migraine			Glycemic traits		Variant annotation	Nearest coding gene
					OR	*P*-value	OR	*P*-value	M-value	OR	*P*-value	M-value		
Migraine and FG														
rs223482	4	103687208	G	A	0.98	5.85 × 10^–11^	0.98	1.05 × 10^–6^	1.00	0.99	2.98 × 10^–7^	1.00	Intergenic	*MANBA
rs6496741	15	91503049	G	A	1.02	1.72 × 10^–9^	1.03	3.07 × 10^–5^	1.00	1.01	5.73 × 10^–7^	1.00	Non-coding transcript exon	*RCCD1*
Migraine and FI														
rs9815656	3	80311982	T	C	0.99	3.24 × 10^–8^	0.98	7.94 × 10^–5^	1.00	0.99	8.59 × 10^–6^	1.00	Intergenic	*ROBO1*
Migraine and Glucose														
rs6658300	1	44129209	T	C	0.98	^a^2.08 × 10^–9^	0.98	1.07 × 10^–5^	1.00	0.99	4.29 × 10^–6^	1.00	Intronic	*KDM4A*
rs4611812	3	64699445	T	C	0.99	1.54 × 10^–10^	0.98	8.78 × 10^–5^	1.00	0.99	1.78 × 10^–7^	1.00	Intronic	*ADAMTS9*
rs9380862	6	39404162	T	C	1.02	2.77 × 10^–9^	1.03	3.40 × 10^–6^	1.00	1.01	1.39 × 10^–5^	1.00	Intronic	*KCNK16*
Migraine and HOMA-B														
rs7944584	11	47336320	T	A	1.02	^a^7.27 × 10^–9^	1.03	6.05 × 10^–6^	1.00	1.02	7.90 × 10^–5^	1.00	Intronic	*MYBPC3*

*SNP* Single nucleotide polymorphism, *CHR* Chromosome, *BP* Base pair position (NCBI37/hg19), *EA* effect allele, *NEA* non-effect allele, *OR* odds ratio for EA, *RE2* random effect; ^a^Results provided for Fixed effect model; *FG* Fasting glucose, *FI* Fasting insulin, *HOMA-B* homeostasis model assessment of β-cell function; M-value: Posterior probability

*These mapped genes are common with the genes that found in our cross-trait meta-analysis of headache and glycemic traits

In the cross-trait meta-analysis of headache and glycemic traits, seven out of the 40 putative novel lead SNPs were found (Supplementary Table S7) to be suggestively associated (*P*_single-trait_ < 1 × 10^–4^) with the single traits GWAS (Table 4). According to the posterior probability (*m*-value > 0.90) estimates, the detected lead SNPs substantially affect both headache and the respective glycemic trait. These seven lead SNPs were mapped to the nearest protein-coding genes of *MANBA, ELFN1, AP3B1, HSPA4, MANBA, NBEAL1,* and *GOLGA6A.* Six of these lead SNPs or loci are novel for both headache and glycemic traits since they were not previously implicated in these traits, while one locus (*MANBA*) being novel for headache but not FG. *ELFN1* gene represented the most significant locus (lead SNP rs28728306, *P*_meta_ = 1.42 × 10^–10^). The second locus was assigned to the *HSPA4* gene (lead SNP rs201681457, *P*_meta_ = 2.81 × 10^–10^), and the third most significant signal was found near the *MANBA* gene (lead SNP rs223482, *P*_meta_ = 3.87 × 10^–10^). Interestingly, a novel locus mapped to the *MANBA* gene was common across the migraine, headache, and glycemic traits. This locus was also identified in the cross-trait GWAS meta-analysis between migraine and FG (lead SNP rs223482), headache and FG (lead SNP: rs223482), and headache and glucose (lead SNP rs223497); and SNPs rs223482 and rs223497 were found to be in high LD (*r*^2^ = 0.85). We also noticed that 10 of the 14 putative novel lead loci revealed in the cross-trait meta-analysis between migraine and headache with glycemic traits were in the genomic region having a posterior probability association (PPA3 > 0.5) in the GWAS-PW association analysis (Supplementary Tables S6 and S7). Supplementary Tables S6 and S7 show the results of cross-trait meta-analysis between migraine and glycemic traits (44 lead SNPs) and headache and glycemic traits (40 lead SNPs) with unadjusted *P* < 0.05 in their individual single-trait GWAS, respectively.

**Table 4:** Cross-trait meta-analysis results for headache and glycemic traits GWAS (*P*_meta_ < 5 × 10^–8^ and single trait *P* < 10^–4^)

Lead SNP	CHR	BP	EA	NEA	RE2 Meta-analysis			Headache			Glycemic traits		Variant annotation	Nearest coding gene
					OR	*P*-value	OR	*P*-value	M-value	OR	*P*-value	M-value		
Headache and FG														
rs223482	4	103687208	G	A	0.98	3.87 × 10^–10^	0.97	7.72 × 10^–6^	1.00	0.99	2.98 × 10^–7^	1.00	Intergenic	*MANBA
rs252768	5	77416148	T	C	0.98	2.57 × 10^–8^	0.97	2.56 × 10^–5^	1.00	0.99	2.30 × 10^–5^	1.00	Intronic	*AP3B1*
Headache and Glucose														
rs223497	4	103653976	C	T	0.98	2.38 × 10^–8^	0.97	9.27 × 10^–6^	1.00	0.99	3.69 × 10^–5^	1.00	Intronic	*MANBA
Headache and HbA1c														
rs375380888	2	203899079	G	A	0.97	6.45 × 10^–10^	0.95	1.19 × 10^–6^	1.00	0.99	1.94 × 10^–6^	0.99	Intronic	*NBEAL1*
rs201681457	5	132402435	C	T	0.98	2.81 × 10^–10^	0.97	5.40 × 10^–7^	1.00	0.99	1.67 × 10^–6^	0.98	Intronic	*HSPA4*
rs28728306	7	1961814	A	C	0.98	1.42 × 10^–10^	0.96	1.44 × 10^–7^	1.00	0.99	2.68 × 10^–6^	0.91	Intronic	*ELFN1*
rs7180506	15	74341208	G	C	0.98	1.73 × 10^–9^	0.97	4.75 × 10^–5^	1.00	0.99	2.49 × 10^–6^	1.00	Downstream gene	*GOLGA6A*

*SNP* Single nucleotide polymorphism, *CHR* Chromosome, *BP* Base pair position (NCBI37/hg19), *EA* effect allele, *NEA* non-effect allele, *OR* odds ratio for EA, *RE2* Random effect; ^a^Results provided for Fixed effect model; *FG* Fasting glucose, *HbA1c* Glycated haemoglobin; M-value: Posterior probability

*These mapped genes are common with the genes that found in our cross-trait meta-analysis of migraine and glycemic traits

### Gene-based association analyses across migraine, headache, and glycemic traits

Supplementary Table S8 displays the findings of binomial tests to assess whether the proportion of genes with small *P* values overlapping migraine and headache with glycemic traits was higher than expected by chance. The primary overlap analysis using gene-based *P* values ≤ 0.05 identified significant evidence of gene-based genetic overlap between migraine and headache with each of the glycemic traits. For instance, the observed proportion of genes overlapping migraine (16.6%) and headache (17%) with FG at *P*_gene_ ≤ 0.05 was significantly higher [*P*_binomial-test_ = 1.24 × 10^–16^ (migraine and FG) and *P*_binomial-test_ = 4.74 × 10^–14^ (headache and FG)] than the expected proportion (11.5% for migraine and FG, and 11.4% for headache and FG), providing additional evidence for highly significant genetic overlap between migraine and headache with FG (Supplementary Table S8 (Table II)). Using both stricter (*P* ≤ 0.01) and less strict (*P* ≤ 0.1) *P* value thresholds also produced evidence for significant gene-based genetic overlap between migraine and each of the glycemic traits, and between headache and each of the glycemic traits (Supplementary Table S8).

At a gene-based genome-wide significant threshold of *P* < (0.05/effective number of independent genes in the gene-based test), 30 genes were overlapping migraine and more than one glycemic trait and 66 genes were overlapping headache and more than one glycemic trait (Tables 5 and 6, respectively). Further details about these genes, including their association *P*-values, are presented in Supplementary Tables S9 and S10. Among these shared genome-wide significant genes between migraine, headache, and glycemic traits, four (*NEU2*, *SLC44A4*, *EHMT2*, and *STAC3*) were common to both migraine and headache that overlapped more than one glycemic trait. Furthermore, five of the 30 genome-wide significant genes overlapping migraine and more than one glycemic trait were the nearest genes to a lead migraine SNP (*THADA*, *EHMT2*, *AMBRA1*, and *SMG6*) (Hautakangas et al. 2022) and headache SNP (*ATG13*) (Meng et al. 2018). In addition, one of the 66 genes (*EHMT2*) overlapping headache and more than one glycemic trait was the nearest gene to a lead migraine SNP (Hautakangas et al. 2022). Using the FCP approach, we calculated the combined *P* values for the genes overlapping migraine and headache with each glycemic trait at *P*_gene_ < 0.05. The results of the FCP show that a total of 483 and 380 unique gene-based genome-wide significant genes overlapping migraine (*P*_FCP_ < 3.64 × 10^–6^ and *P*_single-trait_ < 0.05) and headache (*P*_FCP_ < 3.59 × 10^–6^ and *P*_single-trait_ < 0.05) with more than one glycemic trait, respectively. Among these overlapping genes, 181 were common to migraine and headache that overlapped more than one glycemic trait (Supplementary Tables S11, S12, and S13).

**Table 5:** Genome-wide significant genes associated with migraine and more than one glycemic trait

Genes	Chromosome	Start position (hg19)	End position (hg19)	Glycemic traits
*THADA*	2	43457975	43823185	FG, Glucose, HbA1c
*NEU1	6	31826829	31830709	Glucose, HbA1c, T1D
*SLC44A4	6	31830969	31846823	Glucose, HbA1c, T1D
*EHMT2	6	31847536	31865464	Glucose, HbA1c, T1D
*CALCB*	11	15095143	15103888	Glucose, HbA1c
*AMBRA1*	11	46417962	46615619	FG, Glucose, HbA1c, Proinsulin
*ATG13*	11	46638826	46697569	FG, Glucose, HbA1c, Proinsulin
*ARHGAP1*	11	46698625	46722215	FG, Glucose, HbA1c, Proinsulin
*ZNF408*	11	46722317	46727466	FG, Glucose
*F2*	11	46740743	46761056	FG, Glucose
*CKAP5*	11	46765084	46867859	FG, Glucose, HbA1c, Proinsulin
*LRP4*	11	46878268	46940173	FG, Glucose, Proinsulin
*C11orf49*	11	46958240	47185932	FG, Glucose, HbA1c, Proinsulin
*ARFGAP2*	11	47185849	47199054	FG, Glucose
*PACSIN3*	11	47199073	47208010	FG, Glucose
*DDB2*	11	47236493	47260769	FG, Glucose, HbA1c, Proinsulin
*CELF1*	11	47487489	47574792	FG, Glucose, HbA1c, Proinsulin
*PTPMT1*	11	47586888	47595013	FG, Glucose, HbA1c, Proinsulin
*KBTBD4*	11	47593749	47600567	FG, Glucose, HbA1c
*NDUFS3*	11	47600562	47606115	FG, Glucose, HbA1c
*FAM180B*	11	47608230	47610746	FG, Glucose, HbA1c
*C1QTNF4*	11	47611216	47615961	FG, Glucose, HbA1c, Proinsulin
*MTCH2*	11	47638858	47664206	FG, Glucose, HbA1c, Proinsulin
*RAB3IL1*	11	61664706	61713747	FG, Glucose, HbA1c
*STAC3	12	57637241	57644971	Glucose, HbA1c
*SMG6*	17	1963133	2207069	HbA1c, Proinsulin
*DMWD*	19	46286205	46296060	FG, Glucose, HbA1c
*RSPH6A*	19	46298968	46318605	FG, Glucose, HbA1c
*SYMPK*	19	46318700	46366548	FG, Glucose, HbA1c
*EYA2*	20	45523263	45817492	FI, Glucose

*FG* fasting glucose, *FI* fasting insulin, *Proinsulin* fasting proinsulin, *HbA1c* glycated haemoglobin, *T1D* Type 1 diabetes, *Genes* RefSeq genes, *hg19* human genome version 19

*These genes overlapped with the genome-wide significant genes identified between headache and more than one glycemic trait

**Table 6:** Genome-wide significant genes associated with headache and more than one glycemic trait

Genes	Chromosome	Start position (hg19)	End position (hg19)	Glycemic traits
*ABT1*	6	26597171	26600278	HbA1c, T1D
*ZNF322*	6	26634611	26659980	HbA1c, T1D
*HIST1H2BJ*	6	27093912	27100743	HbA1c, T1D
*HIST1H2AG*	6	27100817	27101314	HbA1c, T1D
*ZNF391*	6	27356524	27371683	HbA1c, T1D
*ZNF184*	6	27418521	27440897	HbA1c, T1D
*HIST1H2BL*	6	27775257	27775709	HbA1c, T1D
*HIST1H2AI*	6	27775977	27776445	HbA1c, T1D
*HIST1H3H*	6	27777842	27778314	HbA1c, T1D
*HIST1H2AJ*	6	27782080	27782518	HbA1c, T1D
*HIST1H2BM*	6	27782822	27783267	HbA1c, T1D
*HIST1H4J*	6	27791903	27792258	HbA1c, T1D
*HIST1H4K*	6	27798952	27799305	HbA1c, T1D
*HIST1H2BN*	6	27805544	27821533	HbA1c, T1D
*HIST1H2AK*	6	27805658	27806117	HbA1c, T1D
*HIST1H2AL*	6	27833107	27833576	HbA1c, T1D
*HIST1H1B*	6	27834570	27835359	HbA1c, T1D
*HIST1H3I*	6	27839623	27840099	HbA1c, T1D
*HIST1H4L*	6	27840926	27841289	HbA1c, T1D
*HIST1H3J*	6	27858093	27858570	HbA1c, T1D
*HIST1H2AM*	6	27860477	27860963	HbA1c, T1D
*HIST1H2BO*	6	27861203	27926100	HbA1c, T1D
*OR2B2*	6	27878963	27880174	HbA1c, T1D
*OR2B6*	6	27925019	27925960	HbA1c, T1D
*ZNF165*	6	28048482	28057341	HbA1c, T1D
*ZSCAN16*	6	28092334	28097864	HbA1c, T1D
*ZKSCAN8*	6	28109688	28127250	HbA1c, T1D
*ZSCAN9*	6	28193029	28201265	HbA1c, T1D
*ZKSCAN4*	6	28212404	28227030	HbA1c, T1D
*NKAPL*	6	28227075	28228736	HbA1c, T1D
*ZSCAN26*	6	28234788	28246001	HbA1c, T1D
*PGBD1*	6	28249314	28270326	HbA1c, T1D
*ZSCAN31*	6	28292514	28321972	HbA1c, T1D
*ZKSCAN3*	6	28317691	28336954	HbA1c, T1D
*ZSCAN12*	6	28346598	28367544	HbA1c, T1D
*ZSCAN23*	6	28399373	28411279	HbA1c, T1D
*C6orf15*	6	31079000	31080332	HbA1c, T1D
*PSORS1C1*	6	31082608	31107869	HbA1c, T1D
*CDSN*	6	31082865	31088252	HbA1c, T1D
*HLA-B*	6	31321649	31324989	HbA1c, T1D
*MICB*	6	31462054	31478901	HbA1c, T1D
*DDX39B*	6	31497996	31510252	HbA1c, T1D
*ATP6V1G2*	6	31512228	31514625	HbA1c, T1D
*NFKBIL1*	6	31514628	31526606	HbA1c, T1D
*LTA*	6	31539876	31542101	HbA1c, T1D
*TNF*	6	31543344	31546113	HbA1c, T1D
*LTB*	6	31548335	31550202	HbA1c, T1D
*LST1*	6	31553956	31556686	HbA1c, T1D
*NCR3*	6	31556660	31560762	HbA1c, T1D
*C6orf25*	6	31691121	31694487	Glucose, HbA1c, T1D
*DDAH2*	6	31694817	31698039	Glucose, HbA1c, T1D
*CLIC1*	6	31698358	31705095	Glucose, HbA1c, T1D
*MSH5*	6	31707725	31730455	Glucose, HbA1c, T1D
*SAPCD1*	6	31730773	31732627	Glucose, HbA1c, T1D
*VWA7*	6	31733178	31745108	Glucose, HbA1c, T1D
*NEU1	6	31826829	31830709	Glucose, HbA1c, T1D
*SLC44A4	6	31830969	31846823	Glucose, HbA1c, T1D
*EHMT2	6	31847536	31865464	Glucose, HbA1c, T1D
*C2*	6	31865562	31913449	Glucose, HbA1c, T1D
*CFB*	6	31913721	31919861	Glucose, HbA1c, T1D
*NELFE*	6	31919864	31926864	Glucose, HbA1c, T1D
*SKIV2L*	6	31926581	31937532	Glucose, HbA1c, T1D
*CYP21A2*	6	32006093	32009447	Glucose, HbA1c, T1D
*TNXB*	6	32008932	32077151	Glucose, HbA1c, T1D
*ATF6B*	6	32083045	32096017	Glucose, HbA1c, T1D
*STAC3	12	57637241	57644971	Glucose, HbA1c

*HbA1c* Glycated haemoglobin, *T1D* Type 1 diabetes, *Genes* RefSeq genes, *hg19* human genome version 19

*These genes overlapped with the genome-wide significant genes identified between migraine and more than one glycemic trait

### Causal analyses between migraine and headache with glycemic traits

**Fig. 2:**
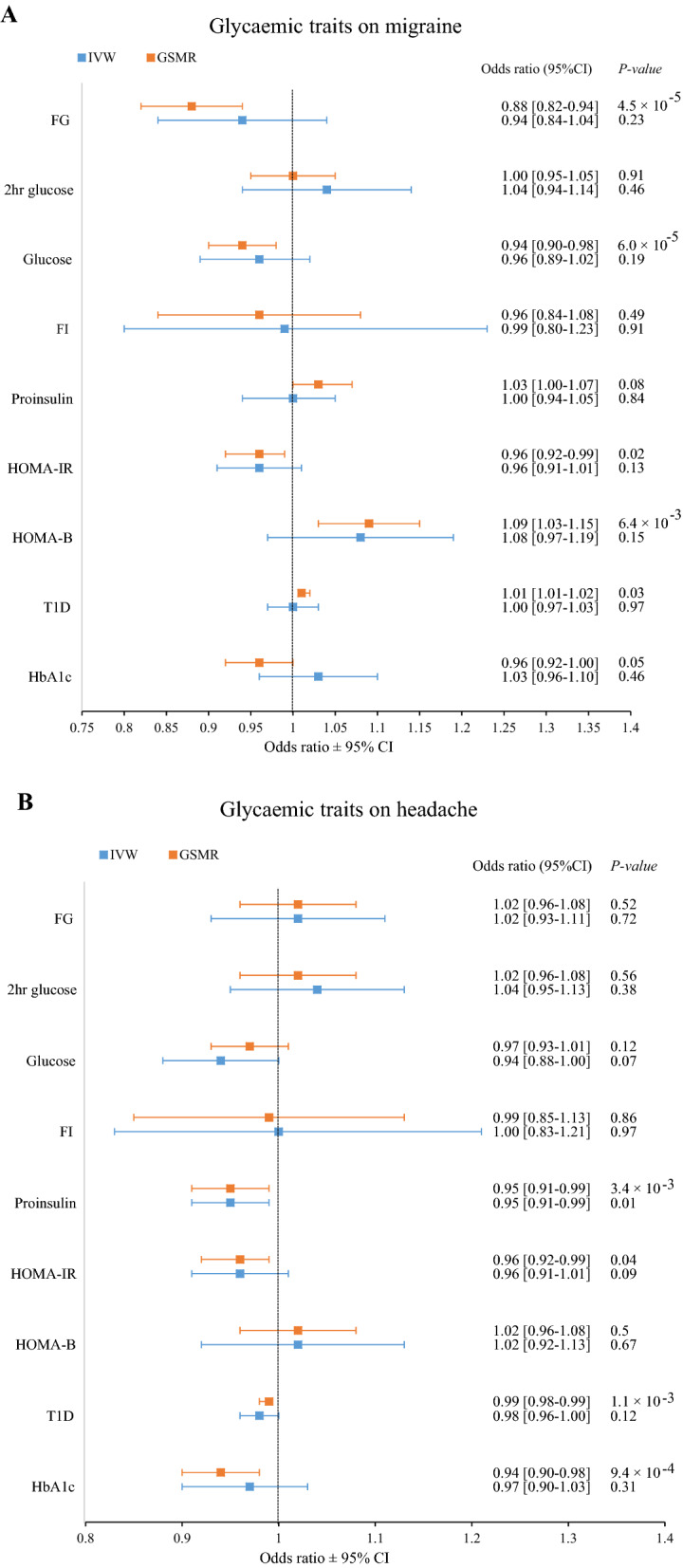
The forest plot demonstrates the odds ratios (ORs) and 95% confidence intervals (CIs) for MR analyses examining the causal effects of glycemic traits on migraine (**A**) and headache (**B**). *IVW* Inverse variance weighted was employed as the primary analysis, *GSMR* Generalised summary data-based Mendelian Randomisation was used as a sensitivity analysis, *FG* Fasting glucose, *FI* Fasting insulin, *HbA1c* Glycated haemoglobin, *HOMA-B* Homeostatic model assessment of β-cell function, *HOMA-IR* Homeostatic model assessment of insulin resistance, *T1D* Type 1 diabetes

Results from the causal analyses between migraine and headache with nine glycemic traits are summarised in Tables 7 and 8, respectively. The primary IVW analyses found that genetic liability to all nine glycemic traits (exposure) was not causally associated with migraine (outcome) (Fig. 2A). A similar pattern of findings was observed for the weighted median, MR-Egger, and MR-PRESSO methods, except for a negative causal effect (protective) of elevated FG on migraine risk estimated by the weighted median method (OR = 0.86 [95% CI 0.77–0.97], *P* = 0.01), although this association did not remain significant after adjusting for multiple testing (Table 7). Interestingly, GSMR analysis also produced that FG has a negative causal effect (OR = 0.88 [95% CI 0.82–0.94], *P* = 4.48 × 10^–5^) on migraine (Table 7). While the MR-Egger intercept (*P* = 0.203) was not significantly different from zero, indicating no significant directional or unbalanced pleiotropy. However, Cochran’s Q statistics for IVW (*P* = 3.76 × 10^–12^) and MR-Egger (*P* = 9.16 × 10^–12^) showed strong heterogeneity among SNPs. Even though MR-PRESSO found three outliers, the effect of removing them was minimal (data not shown). Although the IVW, MR-Egger, and MR-PRESSO analyses did not support these findings, they all agreed with the direction of the results (Table 7). In addition, GSMR analysis revealed evidence indicating that genetic liability to increased HOMA-B (OR_GSMR_ = 1.09, *P* = 6.4 × 10^–3^) and T1D (OR_GSMR_ = 1.01, *P* = 0.03) increases migraine risk, whereas liability to increased glucose (OR = 0.94 [95% CI 0.90–0.98], *P* = 6.0 × 10^–5^), HOMA-IR (OR_GSMR_ = 0.96, *P* = 0.02) and HbA1c (OR_GSMR_ = 0.96, *P* = 0.05) decreases migraine risk; however, these associations were not study-wide significant after adjusting for multiple testing, and primary and other sensitivity MR analyses did not support these findings (Table 7).

**Table 7:** Mendelian randomisation results for migraine and each of the glycemic traits

Exposure	Outcome	nSNPs	IVW		Weighted Median		MR-Egger		MR-PRESSO			nSNPs	GSMR	
			OR (95% CI)	*P*	OR (95% CI)	*P*	OR (95% CI)	*P*	OR	*P*	Global *P*		OR (95% CI)	*P*
FG	Migraine	69	0.94 (0.84–1.04)	0.23	0.86 (0.77–0.97)	**0.01**	0.84 (0.68–1.02)	0.09	0.91	0.07	< 2 × 10^–5^	103	0.88 (0.82–0.94)	**4.48 × 10**^**–5**^
Migraine	FG	104	1.00 (0.99–1.01)	0.46	1.00 (0.99–1.01)	1.00	1.00 (0.97–1.03)	0.89	1.00	0.66	8.6 × 10^–4^	134	1.00 (1.00–1.01)	0.34
2-h glucose	Migraine	14	1.04 (0.94–1.14)	0.46	1.04 (0.96–1.12)	0.36	0.95 (0.73–1.24)	0.71	1.01	0.82	< 2 × 10^–5^	13	1.00 (0.95–1.05)	0.91
Migraine	2-h glucose	104	1.01 (0.96–1.07)	0.6	1.05 (0.98–1.12)	0.14	1.09 (0.95–1.25)	0.22	1.02	0.35	1.6 × 10^–4^	138	1.01 (0.97–1.05)	0.64
Glucose	Migraine	131	0.96 (0.89–1.02)	0.19	0.95 (0.89–1.02)	0.17	1.00 (0.87–1.14)	0.95	0.95	0.10	< 2 × 10^–5^	227	0.94 (0.90–0.98)	**6.0 × 10**^**–5**^
Migraine	Glucose	104	1.00 (0.97–1.02)	0.86	0.99 (0.97–1.01)	0.40	0.96 (0.91–1.01)	0.10	1.00	0.76	< 2 × 10^–5^	134	1.00 (0.98–1.02)	0.49
FI	Migraine	38	0.99 (0.80–1.23)	0.91	1.01 (0.81–1.25)	0.95	0.63 (0.32–1.22)	0.18	-	-	< 2 × 10^–5^	38	0.96 (0.84–1.08)	0.49
Migraine	FI	104	1.01 (1.00–1.02)	0.16	1.00 (0.98–1.01)	0.81	1.01 (0.97–1.04)	0.70	1.01	0.25	< 2 × 10^–5^	132	1.00 (1.00–1.01)	0.33
Proinsulin	Migraine	12	1.00 (0.94–1.05)	0.84	1.02 (0.97–1.07)	0.41	0.98 (0.80–1.21)	0.86	1.02	0.21	< 1 × 10^–3^	14	1.03 (1.00–1.07)	0.08
Migraine	Proinsulin	51	0.99 (0.95–1.04)	0.66	1.04 (0.97–1.11)	0.32	1.07 (0.95–1.21)	0.25	-	-	0.82	119	0.98 (0.94–1.02)	0.28
HOMA-IR	Migraine	66*	0.96 (0.91–1.01)	0.13	0.96 (0.90–1.01)	0.12	0.95 (0.80–1.12)	0.51	-	-	1.4 × 10^–4^	75*	0.96 (0.92–0.99)	**0.02**
Migraine	HOMA-IR	51	1.00 (0.97–1.03)	0.93	0.99 (0.95–1.04)	0.81	1.02 (0.94–1.11)	0.61	-	-	0.05	120	1.00 (0.98–1.02)	0.92
HOMA-B	Migraine	10	1.08 (0.97–1.19)	0.15	1.10 (1.00–1.21)	0.06	1.08 (0.79–1.47)	0.65	1.07	0.18	0.03	11	1.09 (1.03–1.15)	**6.4 × 10**^**–3**^
Migraine	HOMA-B	51	1.01 (0.99–1.04	0.34	1.03 (0.99–1.07)	0.11	1.01 (0.95–1.08)	0.66	-	-	0.11	120	1.02 (1.00–1.04)	0.07
T1D	Migraine	8	1.00 (0.97–1.03)	0.97	1.01 (0.98–1.03)	0.54	1.04 (0.97–1.12)	0.31	1.01	0.41	0.01	48	1.01 (1.01–1.02)	**0.03**
Migraine	T1D	104	1.08 (0.93–1.26)	0.30	1.08 (0.89–1.30)	0.44	0.88 (0.58–1.32)	0.54	1.07	0.30	8 × 10^–5^	139	1.07 (0.95–1.19)	0.21
HbA1c	Migraine	263	1.03 (0.96–1.10)	0.46	0.96 (0.88–1.04)	0.34	0.95 (0.82–1.09)	0.43	1.03	0.42	< 1 × 10^–5^	632	0.96 (0.92–1.00)	0.05
Migraine	HbA1c	104	1.01 (1.00–1.03)	0.09	1.02 (1.01–1.03)	**0.01**	1.01 (0.97–1.05)	0.69	1.01	0.07	< 2 × 10^–5^	123	1.01 (1.00–1.02)	**7.1 × 10**^**–3**^

*IVW* inverse variance weighted, *MR Egger* Egger regression approach, *MR-PRESSO* Mendelian Randomization Pleiotropy RESidual Sum and Outlier, *GSMR* generalized summary data-based Mendelian randomization, *nSNPs* the total number of SNPs used as genetic instruments, *OR* Odds ratio, *CI* Confidence interval, *Global P* Global test *P*-value, *FG* Fasting glucose, *FI* Fasting insulin, *HbA1c* Glycated haemoglobin, *HOMA-B* Homeostatic model assessment of β-cell function, *HOMA-IR* Homeostatic model assessment of insulin resistance, *T1D* Type 1 diabetes

*P < 1 × 10^–5^ threshold used to extract genetic instruments; Dash (–) marked denotes MR-PRESSO produces NAs for outlier corrected analysis if no significant outlier SNPs or horizontal pleiotropy are detected

**Table 8:** Mendelian randomisation results for headache and each of the glycemic traits

Exposure	Outcome	nSNPs	IVW		Weighted Median		MR-Egger		MR-PRESSO			nSNPs	GSMR	
			OR (95% CI)	*P*	OR (95% CI)	*P*	OR (95% CI)	*P*	OR	*P*	Global *P*		OR (95% CI)	*P*
FG	Headache	69	1.02 (0.93–1.11)	0.72	0.99 (0.88–1.11)	0.88	0.95 (0.80–1.13)	0.55	–	–	3.7 × 10^–3^	111	1.02 (0.96–1.08)	0.52
Headache	FG	32	1.00 (0.99–1.02)	0.67	1.01 (0.98–1.03)	0.64	0.98 (0.93–1.03)	0.44	–	–	0.03	40	1.01 (1.00–1.03)	0.45
2-h glucose	Headache	13	1.04 (0.95–1.13)	0.38	1.03 (0.95–1.13)	0.46	1.04 (0.82–1.33)	0.73	1.06	0.12	1.3 × 10^–3^	14	1.02 (0.96–1.08)	0.56
Headache	2-h glucose	32	1.07 (0.99–1.16)	0.07	1.12 (1.01–1.25)	**0.03**	1.08 (0.87–1.34)	0.48	–	–	0.18	39	1.03 (0.97–1.08)	0.32
Glucose	Headache	132	0.94 (0.88–1.00)	0.07	0.99 (0.92–1.06)	0.72	0.96 (0.85–1.09)	0.52	0.96	0.16	< 2 × 10^–5^	248	0.97 (0.93–1.01)	0.12
Headache	Glucose	34	0.98 (0.95–1.01)	0.24	0.98 (0.95–1.01)	0.26	0.93 (0.86–1.02)	0.12	0.99	0.38	< 1 × 10^–4^	39	1.00 (0.98–1.02)	0.66
FI	Headache	38	1.00 (0.83–1.21)	0.97	1.00 (0.81–1.24)	1.00	0.85 (0.49–1.49)	0.57	1.06	0.50	2.2 × 10^–4^	37	0.99 (0.85–1.13)	0.86
Headache	FI	32	1.02 (0.99–1.06)	0.18	0.99 (0.97–1.02)	0.62	1.01 (0.92–1.11)	0.87	1.01	0.28	< 2 × 10^–5^	36	1.01 (0.99–1.03)	0.22
Proinsulin	Headache	12	0.95 (0.91–0.99)	**0.01**	0.95 (0.90–1.00)	0.03	0.85 (0.74–0.98)	**0.05**	–	–	0.12	14	0.95 (0.91–0.99)	**3.4 × 10**^**–3**^
Headache	Proinsulin	19	1.07 (1.00–1.15)	0.06	1.06 (0.96–1.17)	0.23	1.02 (0.84–1.23)	0.87	–	–	0.77	32	1.06 (1.00–1.12)	0.085
HOMA-IR	Headache	66*	0.96 (0.91–1.01)	0.09	0.95 (0.89–1.01)	0.11	1.03 (0.86–1.23)	0.74	–	–	6.2 × 10^–3^	74*	0.96 (0.92–0.99)	**0.041**
Headache	HOMA-IR	19	1.00 (0.96–1.05)	0.90	1.00 (0.94–1.06)	1.00	1.04 (0.92–1.18)	0.54	–	–	0.20	33	0.99 (0.95–1.03)	0.76
HOMA-B	Headache	10	1.02 (0.92–1.13)	0.67	1.03 (0.93–1.14)	0.61	0.92 (0.68–1.25)	0.62	–	–	0.09	11	1.02 (0.96–1.08)	0.50
Headache	HOMA-B	19	1.01 (0.97–1.05)	0.53	1.04 (0.99–1.09)	0.13	1.04 (0.93–1.16)	0.53	–	–	0.09	33	0.99 (0.95–1.03)	0.50
T1D	Headache	9	0.98 (0.96–1.00)	0.12	0.98 (0.97–0.99)	**0.002**	0.98 (0.93–1.03)	0.50	0.98	**0.03**	4 × 10^–3^	43	0.99 (0.98–0.99)	**1.0 × 10**^**–3**^
Headache	T1D	34	1.03 (0.85–1.25)	0.77	1.14 (0.86–1.51)	0.37	1.11 (0.64–1.91)	0.72	–	–	0.73	39	1.08 (0.88–1.28)	0.41
HbA1c	Headache	266	0.97 (0.90–1.03)	0.31	0.98 (0.91–1.06)	0.62	0.92 (0.81–1.05)	0.21	0.97	0.30	< 2 × 10^–5^	662	0.94 (0.90–0.98)	**9.3 × 10**^**–4**^
Headache	HbA1c	34	1.00 (0.96–1.02)	0.66	1.02 (1.00–1.04)	0.10	0.99 (0.91–1.08)	0.85	1.01	0.41	< 2 × 10^–5^	34	1.01 (1.00–1.02)	0.15

*IVW* inverse variance weighted, *MR Egger* Egger regression approach, *MR-PRESSO* Mendelian Randomization Pleiotropy RESidual Sum and Outlier, *GSMR* generalized summary data-based Mendelian randomization, *nSNPs* the total number of SNPs used as genetic instruments, *OR* Odds ratio, *CI* confidence interval, *Global P* Global test *P*-value, *FG* fasting glucose, *FI* fasting insulin, *HbA1c* glycated haemoglobin, *HOMA-B* Homeostatic model assessment of β-cell function, *HOMA-IR* homeostatic model assessment of insulin resistance, *T1D* Type 1 diabetes

*P < 1 × 10^–5^ threshold used to extract genetic instruments; Dash (–) marked denotes MR-PRESSO produces NAs for outlier corrected analysis if no significant outlier SNPs or horizontal pleiotropy are detected

Causal analyses using the IVW model between headache and the nine glycemic traits indicated that increased fasting proinsulin levels reduce the risk of headache (OR = 0.95 [95% CI 0.91–0.99], *P* = 0.01), which was corroborated by all MR sensitivity analyses, although these results were not study-wide significant after adjusting for multiple testing. However, GSMR analysis (OR = 0.95 [95% CI 0.91–0.99], *P* = 3.4 × 10^–3^) did produce significant evidence for a causal relationship between headache and proinsulin (Table 8 and Fig. 2B). The MR-Egger intercept (*P* = 0.16) demonstrated that this was not due to horizontal pleiotropy, and there was no heterogeneity (Cochran’s *Q* *P*_ivw_ = 0.09). In addition, no outlier was found by MR-PRESSO, and the global test (*P* = 0.11, data not shown) was not significant, indicating that there is no horizontal pleiotropy. Additionally, GSMR analysis revealed a significant association between genetic liability to T1D (OR = 0.99 [95% CI 0.98–0.99], *P* = 1 × 10^–3^), HbA1c (OR = 0.94 [(95% CI 0.90–0.98], *P* = 9.4 × 10^–4^), and HOMA-IR (OR = 0.96 [95% CI 0.92–0.99], *P* = 0.04, not significant after adjusting for multiple testing) casually decreases the risk of headache (Table 8). The IVW, Weighted median, MR-Egger, and MR-PRESSO models were not statistically significant; however, the causal estimate was in line with the direction of effect as the GSMR method.

Reverse MR analyses were also performed to test for a causal effect of migraine and headache (exposure) on the nine glycemic traits (outcome) (Tables 7 and 8). Some evidence for a causal association between genetic liability to migraine on HbA1c was provided by GSMR (OR = 1.01 [95% CI 1.01–1.02], *P* = 7 × 10^–3^); however, this association was not significant after adjusting for multiple testing. The weighted median model corroborated this with similar effect sizes; however, the IVW, MR-Egger, and MR-PRESSO models did not provide evidence for a significant causal association of migraine on HbA1c. There was evidence for heterogeneity (Cochran’s *Q* *P*_ivw_ = 1.08 × 10^–43^), although the MR-Egger intercept (*P* = 0.81) indicated that this was most likely not caused by horizontal pleiotropy. There was no significant evidence of migraine having a causal effect on any of the eight remaining glycemic traits. Furthermore, we found no evidence for a causal association between genetic liability to headache on any of the nine glycemic traits. Further details of the genetic instruments utilised for migraine, headache, and glycemic traits in 2SMR (Supplementary Tables S14–S17) and GSMR (Supplementary Tables S18-S19) analysis are presented in the Supplementary Tables.

Although a relatively small SNP-based heritability *z*-score was obtained for fasting proinsulin (*z* < 7), which may lead to an inaccurate heritability estimate, our LCV analyses found significant evidence for partial genetic causality between fasting proinsulin and both migraine (GCP = 0.74, SE = 0.18, *P* = 9.67 × 10^–9^) and headache (GCP = 0.85, SE = 0.11, *P* = 8.05 × 10^–22^) (Supplementary Table S20). No other glycemic traits showed significant evidence for genetic causality with migraine and headache in the LCV analyses. Supplementary Table S20 contains all LCV model results.

### Pathway analysis

Pathway analyses of the genes associated with migraine and the glycemic traits, and headache and the glycemic traits found multiple pathways significantly enriched by the overlapping associated genes (Supplementary Tables S21 and S22). There were no significantly enriched pathways for genes overlapping migraine and HOMA-IR. However, genes associated with migraine and the remaining eight glycemic traits were enriched in pathways including ‘regulation of carbohydrate catabolic process,’ ‘response to axon injury,’ ‘chromatin assembly or disassembly,’ ‘signalling by Notch,’ ‘pre-notch transcription and translation,’ ‘pre-notch expression and processing,’ ‘oxidative stress induced senescence,’ ‘systemic lupus erythematosus,’ and ‘alcoholism’. In addition, genes associated with headache and glycemic traits were enriched in pathways including ‘magnesium ion binding,’ ‘chromatin assembly or disassembly,’ ‘antigen processing and presentation of exogenous peptide antigen,’ ‘nucleosome assembly,’ ‘type I diabetes mellitus,’ ‘autoimmune thyroid disease,’ and ‘systemic lupus erythematosus’ pathways. These pathways may play a role in the mechanisms underlying the co-occurrence of migraine, headache, and glycemic traits. Supplementary Tables S21 and S22 provide further details on these pathways, including the genes involved.

## Discussion

This in-depth genome-wide cross-trait study explores the shared genetic basis underpinning migraine and headache with glycemic traits. We discovered a nominally significant genetic correlation between migraine and five glycemic traits (2-h glucose, FI, fasting proinsulin, T1D, and HbA1c), and between headache and three glycemic traits (FI, fasting proinsulin, and HbA1c). When the genome was divided into independent genomic regions for the pairwise association (GWAS-PW) analysis, we found eight and twelve genomic regions containing significant pleiotropic effects between migraine and glycemic traits, and headache and glycemic traits, respectively. Using cross-trait meta-analysis, we found multiple shared loci between migraine and headache with glycemic traits, and MR analysis indicated a causal role for fasting proinsulin in preventing headache.

In the LDSC analyses, 2-h glucose, FI and HbA1c produced a statistically significant genetic correlation with migraine, while FI and HbA1c produced a significant genetic correlation with a headache. These results offer new insight and are consistent with the known relationship between migraine and higher glucose and insulin levels in published observational epidemiological research (Cavestro et al. 2007; Gruber et al. 2010; Islam and Nyholt 2022; Zhang et al. 2020). In addition, previous research has also discovered that T1D (a high level of HbA1c) and a higher glucose level are inversely associated with migraine (Aamodt et al. 2007; Fagherazzi et al. 2019; Gray and Burtness 1935; Hagen et al. 2018). Our findings, however, show that a shared genetic overlap likely caused the significant association between migraine and headache with glycemic traits. In support of this idea, gene-based association analysis demonstrates a highly substantial amount of gene-level genetic overlap between migraine and headache with glycemic traits.

GWAS-PW analyses found significant shared genomic regions between migraine and glycemic traits, and headache and glycemic traits, including some glycemic traits that did not produce a significant LDSC genetic correlation (Table 2). These pleiotropic regions suggest a shared genetic basis underlying these two traits either directly through the genetic variants that affect both traits because of horizontal pleiotropy and/or vertical pleiotropy, the causal effect of one trait on the other, also known as causality. Among these shared genomic regions, those on chromosomes 1 and 9 harbour the *MEF2D* and *ASTN2* genes, previously reported to be the closest genes to a lead migraine SNP (Hautakangas et al. 2022) and headache SNP (Meng et al. 2018). Interestingly, *ASTN2* (astrotactin 2) is a neuronal protein that plays a crucial function in neural development through its involvement in neuronal adhesion (Jiao et al. 2015). In addition, neuronal pathways can influence insulin sensitivity (Uno et al. 2006) and contribute to the symptoms of insulin resistance syndrome in people with T2D and obesity (Jiao et al. 2015). Earlier research reported that genetic variants within or near the *ASTN2* gene are associated with neuropsychiatric conditions such as schizophrenia, autism, and cognitive dysfunction (Wilson et al. 2010), making it a potential candidate for both migraine and glycemic traits. Additionally, regions at chromosomes 14 and 17 contain the *SERPINA1* and *SMG6* genes, which were previously shown to be the closest genes to a lead migraine SNP (Hautakangas et al. 2022). Notably, there is an overlap between the significant shared regions for migraine and glycemic traits found in chr1: 156336133-158027330, chr2: 209941529-212379238, and chr14: 94325812-95750857 with the shared region reported for headache and glycemic traits. These regions contain genetic variants that were mapped to multiple genome-wide significant genes with top SNPs *P* < 1.0 × 10^–5^ (*C1orf61*, *MEF2D*, *NTRK1*, *CPS1, SERPINA2*, *SERPINA1,* and *ITPK1)*; many of these loci have been previously reported to be the nearest genes to a lead migraine SNP (Hautakangas et al. 2022) and headache SNP (Meng et al. 2018, 2021), thus further highlighting genes that may provide insight into the shared biology underlying the comorbidity of migraine and headache with glycemic traits. The GWAS-PW analyses found no shared genomic regions for HOMA-B and HOMA-IR with migraine and headache; however, given that their GWAS sample sizes are relatively small, further investigation using more powerful HOMA-B and HOMA-IR GWAS summary statistics is required to clarify their relationships.

Genetic correlation estimates the average genetic effects shared between two traits, while GWAS-PW analysis identifies particular genomic regions shared by both traits. We also conducted a cross-trait meta-analysis to explore further the complicated genetic relationships between migraine and headache with glycemic traits. Using cross-trait meta-analysis, we found six novel loci (lead SNPs) shared across migraine and glycemic traits and six novel loci shared across headache and glycemic traits, providing leads into the potential genes and biological pathways underlying their comorbidity (Table 3). Notably, all but one of these 12 novel SNPs (i.e., rs223497 from a meta-analysis of headache and glucose) were identified from a meta-analysis of GWAS datasets with no sample overlap. In terms of potential pathophysiology, among these shared loci, we highlight five interesting loci of *MANBA*, *KCNK16*, *ADAMTS9, NBEAL1*, and *ELFN1*. *MANBA*, a novel locus shared by migraine and FG (lead SNP rs223482), headache and FG (lead SNP: rs223482), and headache and glucose (lead SNP rs223497) in the cross-trait meta-analyses. We also identified the *MANBA* locus as a significant pleiotropic region (chr4: 103221459-105304491) in the GWAS-PW analyses for migraine and FG, headache and FG, and headache and glucose. A study of the *MANBA* expression in brain tissues suggests that the cerebellar cortex, medulla, and pons are the primary sites where it is expressed, in line with the idea that the cerebellar cortex plays a role in migraine pathophysiology (Chen et al. 2022). Guo et al. recently reported *MANBA* as a risk gene associated with migraine and blood pressure through transcriptome-wide association studies (Guo et al. 2020). In addition, genomic regions containing the *MANBA* gene are associated with migraine and small vessel disease (Malik et al. 2015). Several other genes (*CISD2*, *NFKB1*, *SLC9B1/2*, *BDH2*, and *CENPE*) in this region (± 500 kb) have been strongly associated with essential hypertension (Malik et al. 2015), inflammatory diseases, and autoimmune disorders (Lagou et al. 2021); these conditions are significant risk factors for migraine and glycemic traits like hyperglycaemia and IR. However, further research is required to confirm and understand the genetic basis and involvement of *MANBA* in the onset and progression of diseases like migraine and glycemic traits.

*KCNK16*, located near lead SNP rs9380862, was revealed as a significant shared locus associated with both migraine and glucose. *KCNK16* encodes potassium two-pore domain channel subfamily K member 16 (also called TALK1). This channel was shown to be expressed in human and animal pancreatic beta cells and is crucial for the cell’s electrical excitability and glucose-induced insulin secretion (Ndiaye et al. 2017). Reduced expression of the *KCNK16* gene has been suggested to play a role in beta-cell function and was strongly associated with altered insulin secretion (Ndiaye et al. 2017) and discovered as a susceptibility locus for T2D in several GWAS studies conducted in East Asian, Indian, and European descent populations (Cho et al. 2011; Wood et al. 2017). Of the 123 migraine risk loci identified in the most recent and largest GWAS study of migraine (Hautakangas et al. 2022), one ion channel gene (*KCNK5*) was found to be associated with migraine. Additionally, *KCNK16* and *KCNK17* variants were associated with a higher risk of epilepsy as they altered channel currents and spike frequencies (Lee et al. 2021) which is noteworthy given epilepsy is more prevalent in migraine patients than in the general population, and the prevalence of migraine in epilepsy patients is higher than in non-epilepsy controls (Keezer et al. 2015) and migraine and epilepsy have been shown to have a shared genetic etiology (Anttila et al. 2018).

The *ADAMTS9* gene, located near lead SNP rs4611812, encodes the ADAMTS-9 protein, a member of the ADAMTS (a disintegrin and metalloproteinase with thrombospondin motifs) protein family, which may also play a key role in the pathophysiological processes underlying the diseases of the central nervous system like ischemic stroke and spinal cord injury (Lin et al. 2017). Our findings are supported by studies showing the association of the *ADAMTS9* gene with T2D, IR, and insulin sensitivity (Boesgaard et al. 2009; Lin et al. 2017; Zeggini et al. 2008). Given that IR is a risk factor for migraine development, we hypothesised that the genes relevant to IR might be associated with migraine and headache (Islam and Nyholt 2022). Furthermore, in addition to playing a significant role in maintaining normal brain function, insulin imbalances also raise the chance of developing migraines (Islam and Nyholt 2022) and other neurodegenerative diseases like Alzheimer’s and cognitive aging (Lin et al. 2017). However, the molecular mechanisms underlying *ADAMTS9’s* effect on insulin action and migraine risk are currently unknown.

*NBEAL1,* near lead SNP rs375380888, encodes the Neurobeachin-like 1 protein, one of the nine proteins that share the BEACH (Beige and Chediak-Higashi) domain. *NBEAL1* was previously identified in a GWAS of migraine (Gormley et al. 2016) and cerebral small vessel disease (Chung et al. 2019). Furthermore, it was reported to affect cellular cholesterol metabolism and LDL absorption and to be related to coronary artery diseases (Bindesbøll et al. 2020). *NBEAL1* has also recently been found to be associated with white matter hyperintensities (Traylor et al. 2016). However, the biological roles of *NBEAL1* in the pathophysiology of migraine and headache are yet unclear and require further research. *ELFN1* (extracellular-leucine-rich repeat fibronectin domain1), located near lead SNP rs28728306, is abundantly expressed in hippocampal gamma-aminobutyric acid (GABA)-ergic interneurons. A cross-trait genetic study very recently found *ELFN1* implicated in migraine, headache, and T2D (Md Rafiqul Islam et al. 2022). *ELFN1* recruits metabotropic glutamate receptors like mGluR7 to the presynaptic membrane (Stafstrom et al. 2017). *ELFN1-*deficit mutant mice develop seizures (Stafstrom et al. 2017). An imbalance between excitation and inhibition in one or more brain regions causes a seizure. Moreover, neuronal hyperexcitability is also associated with neurological disorders, including migraines and headache, suggesting *ELFN1* to be an exciting candidate gene for migraine susceptibility (Lee et al. 2021).

We further assessed the relationship between migraine and headache with glycemic traits using gene-based association analyses. Our analyses identified shared genes significantly associated with migraine or headache, and multiple glycemic traits. Among the shared genes, four genes (*NEU2*, *SLC44A4*, and *EHMT2* on chromosome 6, and *STAC3* on chromosome 12) were associated with both migraine and headache. Interestingly, neuronal subunits express the protein encoded by the *STAC3* gene, which has Src homology 3 and cysteine-rich domains (Hsu et al. 2020). *STAC3* controls excitation and contraction coupling in murine skeletal muscles and is responsible for a congenital condition known as Native American myopathy (Horstick et al. 2013). *STAC3* has recently been discovered as a risk gene for migraine and metabolic traits (Tanha et al. 2021). Additionally, among the overlapping genes between migraine and headache with glycemic traits, five genes have previously been associated with migraine (*THADA*, *EHMT2*, *AMBRA1*, and *SMG6*) (Hautakangas et al. 2022) and headache (*ATG13*) (Meng et al. 2018); now, these genes are also implicated in glycemic traits. Further research is required to fully understand the function of the shared genes with their putative molecular interactions in the development of migraine, headache, and glycemic traits. Furthermore, the FCP test identified 181 genes with genome-wide significant gene-based *P* values overlapping migraine, headache, and more than one glycemic trait. Among the overlapping genes, four (*ADAMTSL4*, *EHMT2*, *SUGP1*, and *MAU2*) were associated with migraine, headache, and at least five glycemic traits (Supplementary Table S11). Genes like *ADAMTSL4* encode a protease and play a vital role in various biological processes, such as inflammation and angiogenesis (Abramowitz et al. 2016). Interestingly, in addition to migraine (Gormley et al. 2016), *ADAMTSL4* has recently been identified as a risk gene for migraine and cervical artery dissection (Daghals et al. 2022). According to recent research, *MAU2*, a protein-coding gene, has a significant role in chromatin-related functions such as DNA repair and regulation of transcription and has been associated with headache (Meng et al. 2021) and the rare condition Cornelia de Lange Syndrome (Parenti et al. 2020).

Our comprehensive MR investigation results support the hypothesis that a genetic liability to fasting proinsulin may reduce the risk of headache. The sensitivity analysis likewise supports this finding, indicating that horizontal pleiotropy is unlikely to impact the outcomes. This conclusion is further corroborated by LCV, which found a partial causal relationship between fasting proinsulin and headache. We do not currently understand the pathophysiological mechanisms that may underlie a protective effect of fasting proinsulin on headache. However, proinsulin has been identified to activate neuroprotective pathways in the hippocampus and trigger a downstream signalling cascade that reduces astrocyte reactivity and neuroinflammatory biomarkers, all of which are involved in the pathogenesis of migraine, headache, and other neurological conditions (Corpas et al. 2017). Additionally, we note that many significant genes (e.g., *AMBRA1*, *ATG13*, *ARHGAP1*, *CKAP5*, *LRP4*, and *DDB2*; Supplementary Tables S12 and S13) have been associated with migraine, headache, and proinsulin. Intriguingly, *AMBRA1* is involved in many cellular processes, including neuroprotection, apoptosis, autophagy, and neuronal development (La Barbera et al. 2019). GWAS have shown that *AMBRA1*, along with other ATG proteins like *ATG13* (autophagy-related 13), is involved in activating beclin-1-regulated autophagy and favours the autophagosome core complex (Portilla-Fernandez et al. 2019). In experimental settings, disturbed proinsulin or insulin homeostasis in pancreatic beta cells has also been associated with a dysfunctional autophagy process (Portilla-Fernandez et al. 2019). Recent evidence suggested that abnormal autophagy is involved in neuropathic pain and may have a role in the etiology of chronic migraine by controlling microglial activation and following inflammatory response (Jiang et al. 2021). It supports the idea that autophagy pathways may be involved in the association of proinsulin with migraine and headache. Further research is needed to determine which specific function of proinsulin may reduce the risk of headache. Thus, deciphering the molecular mechanisms behind proinsulin’s protective effect on headache should be of interest to the headache research community.

Additionally, we note inconclusive evidence for the causal effects of some of the glycemic traits on migraine and headache after adjusting for multiple testing, primarily in GSMR analysis, although the direction of causal effects was consistent across various MR sensitivity analyses. For instance, we found evidence that genetic liability to FG and glucose causally reduce migraine; however, our sensitivity studies revealed that this finding could have been affected by other biological processes because of horizontal pleiotropy. Similarly, there was inconsistent evidence that genetic liability to HbA1c was associated with a decreased risk of headache. Interestingly, these results support earlier observational findings that hypoglycaemia triggers migraines (Gray and Burtness 1935), and hyperglycaemia or a higher level of HbA1c can prevent migraine headache (Fagherazzi et al. 2019; Hagen et al. 2018). These differences in MR findings may be due to the lower number of genetic instruments utilised in the 2SMR analysis than in the GSMR analysis, hence there may be inadequate power to detect a causal relationship across all methods. Additional research with more powerful GWAS summary statistics is required to investigate the relationship between markers of glycemic dysregulation and the risk of migraine and headache. Given none of the other glycemic traits were causally associated with migraine and headache risk in the MR analyses, their relationships appear most likely to be due to shared genetics (pleiotropy) and common biological mechanisms rather than causality.

Gene-based association analyses of migraine and headache with glycemic traits identified significant overlapping genes enriched in multiple pathways such as cell signalling (e.g., signalling by a notch), cellular processes, oxidative stress-induced senescence, epigenetic mechanisms, catabolic and autoimmune pathways. According to earlier observational studies (Biscetti et al. 2021; Borkum 2016; Gerring et al. 2018; Gross et al. 2019), there is a relationship between metabolic imbalances, oxidative stress, epigenetics, and autoimmunity with migraine and headache. Our results thus support the potential involvement of these pathways in the comorbidity of migraine and headache with glycemic traits.

### Strengths and limitations

Our research has several important strengths. First, we conducted robust cross-trait analyses leveraging the most comprehensive GWAS summary statistics and current statistical genetic methodologies. In addition to estimating genetic correlations and identifying shared loci, we used the LCV technique and multiple 2SMR approaches, including GSMR, to test for causality, which combined, should minimise the chance of false positive causal relationships and avoid bias due to weak instruments, reverse causality, and horizontal pleiotropy. However, we acknowledge several limitations in our study. First, the generalisability of our findings is limited to European ancestry populations; however, we could not analyse non-European populations due to the unavailability of summary statistics from migraine and headache GWAS in non-European samples. Therefore, when they become available, it will be important to conduct analogous cross-trait analyses using GWAS summary statistics from non-European ancestries. Second, although we analysed the latest and most powerful GWAS summary statistics, there are currently no sufficiently-powered independent migraine and headache GWAS datasets that could be used to replicate our results. Third, the GWAS summary statistics for fasting proinsulin are limited by their relatively small sample size; therefore, larger and more powerful GWAS summary statistics for fasting proinsulin are needed to conclusively characterise a causal relationship between migraine and headache. Fourth, examination of LDSC gcov_int values indicated a small sample overlap between headache and glucose and headache and T1D. Although the levels of sample overlap are unlikely to confound the cross-trait SNP meta-analyses, future analyses should utilize non-overlapping samples to ultimately confirm the relevant findings. Fifth, we acknowledge that there were not many IVs for some glycemic traits (i.e., HOMA-B, HOMA-IR, and T1D) at a genome-wide significant level in our MR analyses. Thus, further investigations are warranted using future, more powerful GWAS summary statistics (producing stronger genetic instruments) for HOMA-B, HOMA-IR, and T1D.

## Conclusion

The current study confirmed and provides an improved understanding of the relationship between migraine, headache, and glycemic traits by identifying genetic correlation, and shared loci and genes, and inferring causal association. These findings provide novel insight into the potential underlying biology influencing migraine, headache, and glycemic traits. The implicated genes, loci, and pathways, provide important targets for further functional investigations to uncover the precise molecular mechanisms that contribute to their comorbidity. Furthermore, findings from this study provide important motivation and avenues to develop novel treatment strategies for managing glycemic traits in migraine and headache patients, particularly increasing fasting proinsulin levels to protect against headache.

## Supplementary Information

Below is the link to the electronic supplementary material.

Supplementary file1 (XLSX 1190 KB)

## Data Availability

The data from the MAGIC consortium and Pan-UKBB are available on their respective websites. The migraine GWAS summary statistics are obtained from the 23andMe and IHGC. The full GWAS summary statistics for the 23andMe discovery data set will be made available through 23andMe to qualified researchers under an agreement with 23andMe that protects the privacy of the 23andMe participants. For further details and to access the data, please visit https://research.23andme.com/collaborate/#dataset-access/. Researchers can perform a meta-analysis of 23andMe migraine summary statistics and IHGC migraine GWAS summary statistics to get the full summary statistics of the migraine dataset.
